# The body mass index and the risk of ectopic pregnancy: a 5-year retrospective case-control study

**DOI:** 10.1186/s12884-024-06319-z

**Published:** 2024-02-17

**Authors:** Jin-Shuang Ji, Ling Liu, Huan Huang, Hong-Wei Chen, Li Xiao, Xiang-Yi Lu, Yang-Yang Ni, Wen-Juan Jia, Lei Huang

**Affiliations:** 1 Department of Gynecology & Obstetrics, Tongji Medical College, The Central Hospital of Wuhan, Huazhong University of Science and Technology, Wuhan, 430014 Hubei China; 2 The Diagnosis and Therapy Center of Pelvic Floor Rehabilitation and Electrophysiology, Tongji Medical College, The Central Hospital of Wuhan, Huazhong University of Science and Technology, Wuhan, 430014 Hubei China

**Keywords:** Ectopic pregnancy (EP), Risk factors, Low body mass index (BMI), Retrospective case-control study

## Abstract

**Purpose:**

Acknowledging the associated risk factors may have a positive impact on reducing the incidence of ectopic pregnancy (EP). In recent years, body mass index (BMI) has been mentioned in research. However, few studies are available and controversial on the relationship between EP and BMI.

**Methods:**

We retrospectively studied the EP women as a case group and the deliveries as a control group in the central hospital of Wuhan during 2017 ~ 2021. χ^2^ test of variables associated with ectopic pregnancy was performed to find differences. Univariate and multivariate binary logistic regression analysis was conducted to analyze the association of the variables of age, parity, history of induced abortion, history of ectopic pregnancy, history of spontaneous abortion, history of appendectomy surgery and BMI (< 18.5 kg/m^2^, 18.5 ~ 24.9 kg/m^2^, 25 kg/m^2^ ~ 29.9 kg/m^2^, ≥ 30 kg /m^2^) with EP.

**Results:**

They were 659 EP and 1460 deliveries. The variables of age, parity, history of induced abortion, history of ectopic pregnancy and BMI were different significantly(*P* < 0.05). Multivariate analysis showed that the variables of age > 35 years old [(OR (Odds Ratio), 5.415; 95%CI (Confidence Interval), 4.006 ~ 7.320, *P* < 0.001], history of ectopic pregnancy (OR, 3.944; 95%CI, 2.405 ~ 6.467; *P* < 0.001), history of induced abortion(OR, 3.365; 95%CI, 2.724 ~ 4.158, *P* < 0.001) and low BMI (< 18.5 kg/m^2^) (OR, 1.929; 95%CI, 1.416 ~ 2.628, *P* < 0.001])increased the risk of EP.

**Conclusion:**

The history of ectopic pregnancy, history of induced abortion and age > 35 years old were the risk factors with EP. In addition to these traditional factors, we found low BMI (< 18.5 kg/m^2^) with women may increase the risk to EP.

## Introduction

Ectopic pregnancy (EP) refers to a pregnancy that occurs outside the uterine cavity, and the incidence rate accounts for about 1%~2% of all pregnancies [1, 2]. More than 90% of EP are tubal pregnancy accounting for the first maternal mortality rate in first-trimester pregnancy [3, 4]. Nowadays, the incidence of ectopic pregnancy is still on the rise worldwide [5–7]. We have known that the risk factors for EP, including the history of ectopic pregnancy, the history of tubal surgery, the history of induced abortion, the history of spontaneous abortion, chlamydial infection and pelvic inflammatory disease, the history of infertility, the history of smoking, age > 35 years old [8–12]. With the growth of economic level and the improvement of people’s living standard, risk factors for ectopic pregnancy are also in change.

In recent years, due to aesthetic aberrations, more and more women pursued the ideal body or were underweight [13, 14]. And in clinical workflow, we observed patients with ectopic pregnancies were lean. Whether there is some association between body mass index (BMI) and ectopic pregnancy. In a prospective study, it suggested that low BMI was associated with EP after receiving assisted reproductive technology (ART) [15]. Another study suggested that obese women were at higher risk of ectopic pregnancy [16]. However, BMI may not be associated with ectopic pregnancy in other studies [11, 17]. At present, there are different opinions on the relationship between BMI and EP. The findings of these studies are conflicting, and the studies on BMI did not control other related risk factors. So, there is some bias. There are few studies on the direct relationship between BMI and EP, and further research is needed to confirm their relationship.

This study was retrospective case-control research in the central hospital of Wuhan during 2017 ~ 2021. We included BMI in the study of risk factors. We performed univariate and multivariate binary logistic regression analysis to find relationship between BMI and EP. We found a new risk factor for ectopic pregnancy. It alerts and advocates women to have a healthy body mass index to protect fertility.

## Methods

### Study population

We retrospectively studied the case of ectopic pregnancy (EP) as a case group in the central hospital of Wuhan during 2017 ~ 2021. And pregnant women who gave birth and filed in this hospital during the same period were randomly selected as the control group (according to the order of registration, 10 records were randomly selected from each page). Prepregnancy height (m), weight (kg) and other mask data were provided by the medical information department of the hospital and filing system. This study had been approved by the institutional ethics committees of the central hospital of Wuhan.

**Fig. 1:**
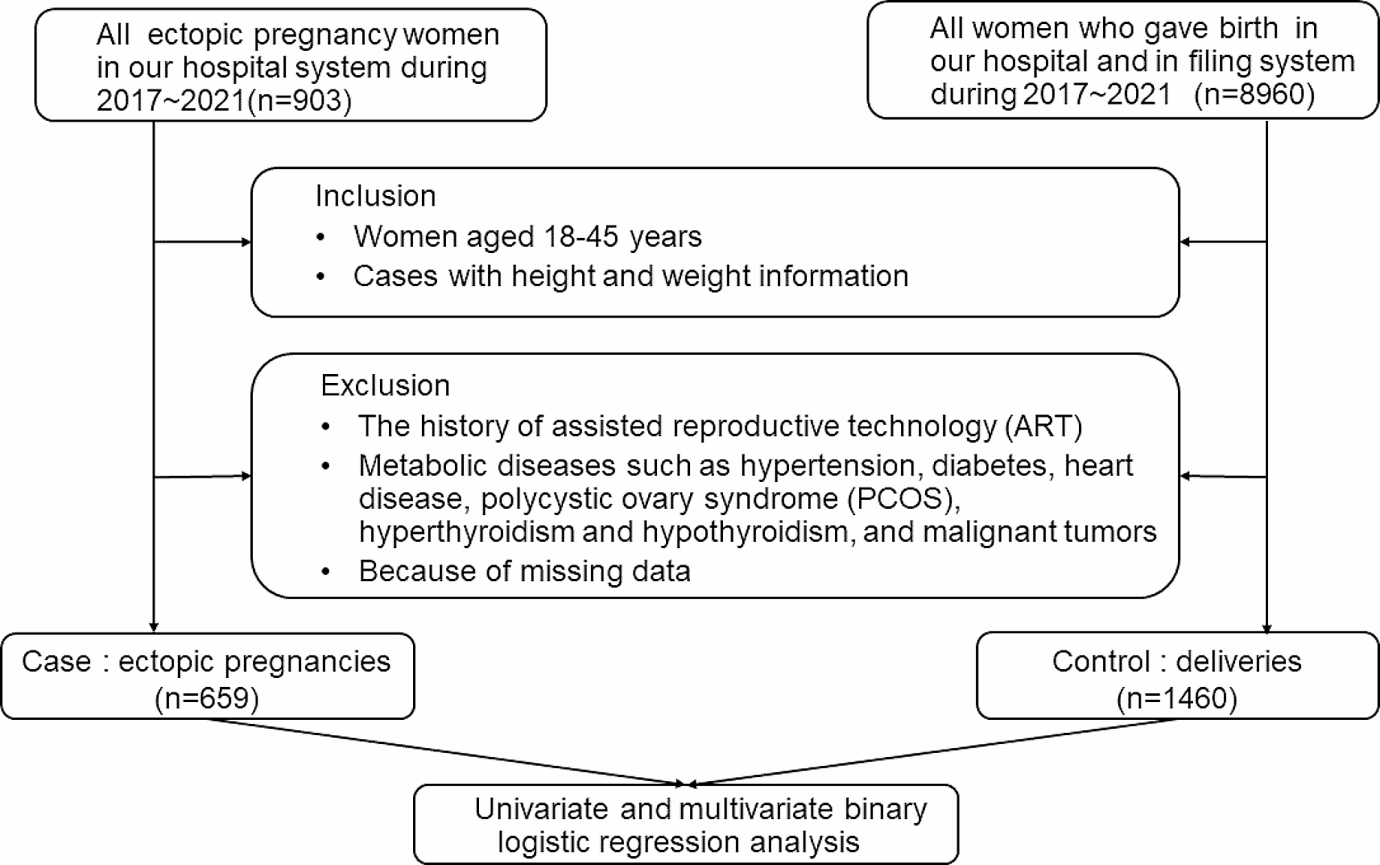
The flowchart of the study

Diagnosis of ectopic pregnancy: confirmed by laparoscopic surgery and pathological diagnosis. A retrospective cohort study was conducted in the central hospital of Wuhan between January 2017 and December 2021. Inclusion criteria: Women aged 18–45 years, Cases with height and weight information. Exclusion criteria: The history of assisted reproductive technology (ART), those with metabolic diseases such as hypertension, diabetes, heart disease, polycystic ovary syndrome (PCOS), hyperthyroidism and hypothyroidism, and malignant tumors, because of missing data. According to the inclusion and exclusion criteria, the case group and control group were obtained. The flowchart of the study was in (Fig. 1).

### Study variables

Sociodemographic characteristics, including age, occupation and region. For comparability between ectopic pregnancy and delivery, we included the previously known risk factors of EP, these variables were age, parity, history of induced abortion, history of ectopic pregnancy, history of spontaneous abortion, history of appendectomy surgery and BMI. According to the World Health Organization (WHO) of classification standard of BMI in 2021, it is divided into low BMI (< 18.5 kg/m^2^), normal BMI (18.5 ~ 24.9 kg/m^2^), overweight (25 ~ 29.9 kg/m^2^), obesity (≥ 30 kg /m^2^). BMI is defined as the body weight divided by the square of height.

### Study outcomes

The primary outcomes of this study were age > 35 years old, history of induced abortion, history of ectopic pregnancy, low BMI (< 18.5 kg/m^2^).

### Statistical analysis

Categorical variables were expressed as frequencies and percentages, and χ^2^ test was used to assess the difference of variables. Binomial logistic regression was for univariate and multivariate analyses.

IBM SPSS Statistics (R26.0.0.0) software was used for data analysis and GraphPad prism (R9.3.1) software for graphing. All comparisons were two-tailed. When *P* value < 0.05, the results were considered statistically significant.

## Results

### Sociodemographic characteristics of women

The sociodemographic characteristics of age, region and occupation in the 2119 women were in Table 1. The results showed that there were 659 EP in the case group and 1460 deliveries in the control group. There were significant differences in age and occupation between groups (*P* < 0.001). The median age of EP patients were 31(27 ~ 36) years old. In these women, 949 (46.2%) women were employees, and 272 (42.2%) patients with EP were employees. region distribution showed no significant differences between groups (*P* = 0.418).

**Table 1:** Sociodemographic characteristics of 2119 women

	Total (%)	Deliveries (%)	EP^a^ (%)	**P**
	(*n* = 2119)	(*n* = 1460)	(*n* = 659)	
Age		29(27 ~ 32)	31(27 ~ 36)	< 0.001
region				0.418
urban	1407(68.10)	986(67.5)	421(69.4)	
rural	660(31.90)	474(32.5)	186(30.6)	
Occupation				< 0.001
Unemployed	157(7.6)	28(2.0)	129(20.0)	
Freelance	530(25.8)	463(32.8)	67(10.4)	
employee	949(46.2)	677(48.0)	272(42.2)	
Self-employed	43(2.1)	8(0.6)	35(5.4)	
The medical health care sector	159(7.7)	125(8.9)	34(5.3)	
others	217(10.6)	109(7.7)	108(16.7)	

^a^EP was represented for ectopic pregnancy

### Analysis for the clinical characteristics of ectopic pregnancy

We included risk factors associated with ectopic pregnancy, then performed chi-square test to compare these variables. The results showed that history of spontaneous abortion and history of appendectomy surgery was not statistically significant(*P* > 0.05). However, the variables of age, parity, history of induced abortion, History of ectopic pregnancy and BMI were different significantly(*P* < 0.001). 498(75.6%) patients with EP were normal BMI. 89(13.5%) EP was low BMI. 59(9.0%) patients with EP were overweight. 13(2.0%) EP was obesity. In summary, the majority of patients (75.6%) of EP was normal BMI, secondly 13.5% patients with EP were low BMI (Table 2).

**Table 2:** The analysis of the difference to the variables of ectopic pregnancy

Variables	Total (*n* = 2119)	Group		**P**
		Control^a^(*n* = 1460)	Case^b^(*n* = 659)	
Age				< 0.001
≤ 35	1868(88.2%)	1378(94.4%)	490(74.4%)	
> 35	251(11.8%)	82(5.6%)	169(25.6%)	
Parity				0.003
0	1530(72.2%)	1083(74.2%)	447(67.8%)	
≥ 1	589(27.8%)	377(25.8%)	212(32.2%)	
History of induced abortion				< 0.001
NO	1409(66.5%)	1107(75.8%)	302(45.8%)	
YES	710(33.5%)	353(24.2%)	357(54.2%)	
History of spontaneous abortion				0.175
NO	1999(94.3%)	1384(94.8%)	615(93.3%)	
YES	120(5.7%)	76(5.2%)	44(6.7%)	
History of ectopic pregnancy				< 0.001
NO	2031(95.8%)	1432(98.1%)	599(90.9%)	
YES	88(4.2%)	28(1.9%)	60(9.1%)	
History of appendectomy surgery				0.569
NO	2068(97.6%)	1423(97.5%)	645(97.9%)	
YES	51(2.4%)	37(2.5%)	14(2.1%)	
BMI				0.004
< 18.5	229(10.8%)	140(9.6%)	89(13.5%)	
18.5 ~ 24.9	1700(80.2%)	1202(82.3%)	498(75.6%)	
25 ~ 29.9	154(7.3%)	95(6.5%)	59(9.0%)	
≥ 30	36(1.7%)	23(1.6%)	13(2.0%)	

^a^control was the deliveries, ^b^case was the ectopic pregnancies

### Univariate and multivariate analysis of risk factors of ectopic pregnancy

We performed univariate and multivariate binomial logistic regression analysis to find risk factors of EP. univariate analysis showed that the variables of age, parity, history of induced abortion, history of ectopic pregnancy and BMI were risk factors of EP (*P* < 0.001). The history of spontaneous abortion was the risk factor with EP and but not statistically significant (*P* = 0.176). the history of appendectomy surgery was the protective factor with EP and but not statistically significant (*P* = 0.569).

**Fig. 2:**
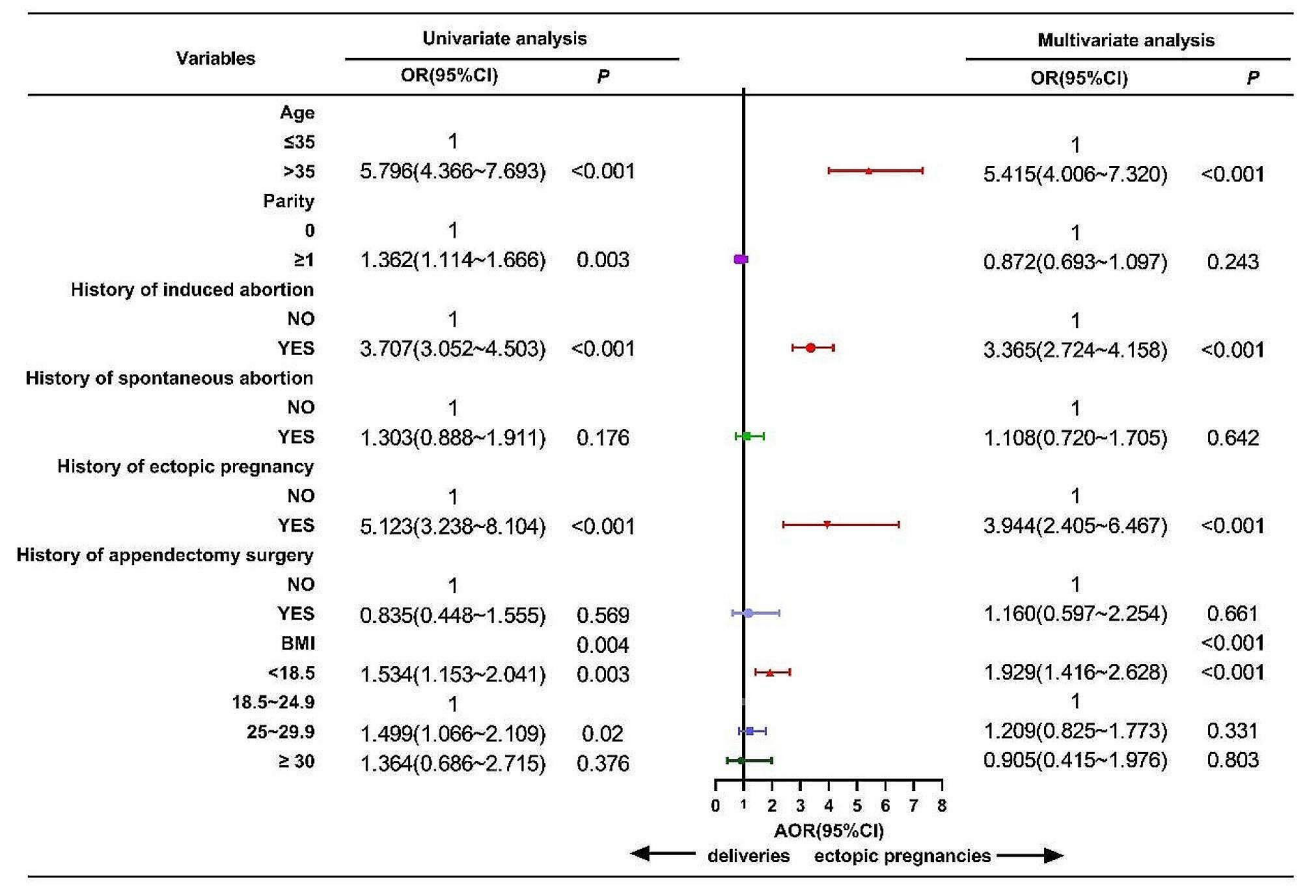
Forest plot of univariate and multivariate of binomial logical regression analysis of EP

Multivariate analysis revealed that the variables of age, history of induced abortion, history of ectopic pregnancy and BMI were risk factors for EP(*P* < 0.001). according to the value of Odds Ratio from large to small size was age > 35 years old [OR, 5.415; 95%CI, 4.006 ~ 7.320, *P* < 0.001], history of ectopic pregnancy [OR, 3.944; 95%CI, 2.405 ~ 6.467; *P* < 0.001], history of induced abortion[(OR, 3.365; 95%CI, 2.724 ~ 4.158, *P* < 0.001], low BMI (< 18.5 kg/m^2^) [(OR, 1.929; 95%CI, 1.416 ~ 2.628, *P* < 0.001].And yet, Overweight (25 kg/m^2^ ~ 29.9 kg/m^2^) was the risk factor of EP but no statistically significant (*P* = 0.331), obesity(≥ 30 kg/m^2^) was the protective factor with EP insignificantly (*P* = 0.803) (Fig. 2).

## Discussion

In this study, we directly included risk factors associated with ectopic pregnancy as well as body mass index to conduct univariate and multivariate analysis. This is a relatively systematic study of risk factors for ectopic pregnancy, a new variable of body mass index was also included. We found the history of ectopic pregnancy, history of induced abortion, age > 35 years old and low BMI (< 18.5 kg/m^2^) were the risk factors with EP. And low BMI (< 18.5 kg/m^2^) was 1.929 times higher risk to EP compared with normal BMI (18.5 kg/m^2^ ~ 24.9 kg/m^2^). Our findings hope that it may improve awareness of these factors, and further research common to these conditions.

Theoretically, any condition that prevents or retards migration of the fertilized ovum to the uterus could predispose a woman to ectopic gestation (current intrauterine device use, the history of infertility, the history of pelvic inflammatory disease, and prior tubal surgery) [18]. As the economy develops, more and more women choose the contraceptive method of IUD. The risk of ectopic pregnancy in different types of intrauterine device may be different, and the concentration of drugs in IUDs may have some mechanism of action with fallopian tube function. In addition, IUDs are foreign to the uterine cavity and may cause the endometrium to be out of sync with fallopian tube function [19, 20]. Pelvic inflammatory disease (PID) changes the inner environment of the pelvic uterus, affecting the transport environment of fallopian tubes, thereby increasing the risk of ectopic pregnancy. The two most common pathogens, Neisseria gonorrhoea and chlamydia trachomatis, have been shown to be mainly associated with ectopic pregnancy [21–23]. Whether the inflammation caused by appendicitis also affects the function of fallopian tubes, there is currently no evidence of higher levels. However, some retrospective studies have shown that the history of appendectomy is associated with increasing risk of ectopic pregnancy [24]. In contrast to our study, we found that women with previous history of appendectomy were not at increased risk of ectopic pregnancy, either through univariate analysis or and multivariate analysis. So, the problem is whether abdominal surgery or inflammation of the appendix caused the ectopic pregnancy. Similarly, the history of two or three cesarean deliveries is associated with increased risk for subsequent ectopic pregnancy in relation to women who had one prior cesarean delivery [25]. We hypothesize that multiple prior cesareans may increase pelvic adhesion leading to tubal hypoplasia. However, there is no experimental evidence.

With age, some risk factors associated with ectopic pregnancy may accumulate. Epidemiological surveys showed a worldwide phenomenon of postponement of the age of childbearing to the 30s, fertility rates were also declining [26]. With the improvement of economic and educational level, the divorce rate and multiple sexual partners among these women are likely to increase [27]. This may lead to pelvic inflammatory disease and tubal disease, and ectopic pregnancy. In a Chinese study, the proportion of EP among those ≥ 35 years old was reversed from a downward trend (2011–2016 annual percentage change (APC) − 4.13) to an upward trend (2016–2020 APC 4.04) [28]. Some study showed that age > 35 years increased the risk of ectopic pregnancy [29, 30]. In agreement with our data, ectopic pregnancy was more likely to occur over the age of 35, the value of odds ratio was 5.415 and the largest in this study, there was a major impact effect on ectopic pregnancy.

There has been a great deal of research showing that an increased risk of repeat EP in patients with history of ectopic pregnancies. In a large French case-control study, women with one ectopic pregnancy had the higher risk of repeated ectopic pregnancy (OR = 12.5), especially for women with two or more ectopic pregnancies [9]. In a recent study, women with the history of ectopic pregnancy had the 2.72 times higher risk of ectopic pregnancy recurrence (Adjusted odds ratio [AOR] = 2.72, 95% confidence interval [CI]: 1.83–4.05) [19]. In our study, multivariate analysis showed that the risk of ectopic pregnancy was 3.944 times higher in women with the history of ectopic pregnancy. Women with previous history of ectopic pregnancy may affect fallopian tube function after conservative or surgical treatment, such as oviduct blockage, oviduct water accumulation, oviduct inflammation, etc.

Induced abortion remains noticeable in China [31]. In a recent cross-sectional study, of all abortions, 65.2% were repeat induced abortions [32]. The history of induced abortion was associated with an increased risk of ectopic pregnancy [OR, 1.5; 95%CI, 1.0 ~ 2.0], particularly in the case of women who have had several induced abortions [33]. In a prospective study by Skjeldestad, Induced abortion did not increase the risk of ectopic pregnancy [34]. In our study, the history of induced abortion was significantly associated with ectopic pregnancy(OR, 3.365; 95%CI, 2.724 ~ 4.158). But the relationship between history of spontaneous abortion and ectopic pregnancy was no statistically significant. We assumed that one or more abortions increased the chance of uterine manipulation, leading to an enhanced risk of uterine infection. The possibility of pelvic inflammatory disease and tubal disease cannot be excluded.

In this study, we considered the inclusion of body mass index to explore the risk factors for ectopic pregnancy. However, the relationship between BMI and EP is rarely studied. Pan pointed out that obese women have a higher risk of EP [16], and it was suggested that low BMI was associated with EP after receiving ART in this prospective study [15]. In these studies, the relationship between BMI and EP was not directly investigated, and other factors associated with EP were not controlled. Therefore, there was difference. In our study, by including risk factors associated with EP, multivariate analysis showed that body mass index was associated with EP after adjustment and appeared in low BMI (< 18.5 kg/m^2^). There were few reports in the literature on the mechanism. We consider that leptin levels in women with low body mass index are alternately regulated with insulin growth factor-1 [35, 36], and that leptin is critically linked to reproductive function [37]. However, a unique hormonal regulation exists during embryonic development, maturation, and egg transport through the fallopian oviduct [38]. This suggests that it may affect embryonic development and fallopian tube function by impacting hormonal regulation.

This study is a retrospective study, which effect is less than randomized controlled studies. Subsequent multicenter trial and large-sample research is required. The BMI is a new risk factor compared to other factors, which is a highlight of our research. We are better able to regulate weight relative to other physiological factors to guide the clinical.

## Conclusion

The history of ectopic pregnancy, history of induced abortion and age > 35 years old were the risk factors with EP. In addition to the traditional risk factors, we found an association between body mass index and the risk of ectopic pregnancy. Women with a low BMI (< 18.5 kg/m^2^) had a slightly higher risk of ectopic pregnancy than women with normal BMI. We hope future studies focus on these risk factors and advocate a healthy body mass index to protect female fertility by improving body mass index.

## Data Availability

The datasets used and/or analyzed during the current study are available from the corresponding author on reasonable request.

## Abbreviations

EP: Ectopic pregnancy
BMI: Body mass index

## References

[1] (CDC) CfDCaP (1995). Ectopic pregnancy–United States, 1990–1992. MMWR Morb Mortal Wkly Rep.

[2] Marion LL, Meeks GR (2012). Ectopic pregnancy: history, incidence, epidemiology, and risk factors. Clin Obstet Gynecol.

[3] Khan KS, Wojdyla D, Say L, Gülmezoglu AM, Van Look PF (2006). WHO analysis of causes of maternal death: a systematic review. Lancet.

[4] Bulletins—Gynecology ACoOaGCoP (2018). ACOG Practice Bulletin 193: Tubal ectopic pregnancy. Obstet Gynecol.

[5] Assouni Mindjah YA, Essiben F, Foumane P, Dohbit JS, Mboudou ET (2018). Risk factors for ectopic pregnancy in a population of Cameroonian women: a case-control study. PLoS ONE.

[6] Kopp-Kallner H, Linder M, Cesta CE, Segovia Chacón S, Kieler H, Graner S (2022). Method of Hormonal Contraception and Protective effects against ectopic pregnancy. Obstet Gynecol.

[7] Tay JI, Moore J, Walker JJ (2000). Ectopic pregnancy. BMJ.

[8] Barnhart KT, Sammel MD, Gracia CR, Chittams J, Hummel AC, Shaunik A (2006). Risk factors for ectopic pregnancy in women with symptomatic first-trimester pregnancies. Fertil Steril.

[9] Bouyer J, Coste J, Shojaei T, Pouly J-L, Fernandez H, Gerbaud L, Job-Spira N (2003). Risk factors for ectopic pregnancy: a comprehensive analysis based on a large case-control, population-based study in France. Am J Epidemiol.

[10] Reekie J, Donovan B, Guy R, Hocking JS, Kaldor JM, Mak D, Preen D, Ward J, Liu B (2019). Risk of ectopic pregnancy and Tubal Infertility following Gonorrhea and Chlamydia infections. Clin Infect Dis.

[11] Gaskins AJ, Missmer SA, Rich-Edwards JW, Williams PL, Souter I, Chavarro JE (2018). Demographic, lifestyle, and reproductive risk factors for ectopic pregnancy. Fertil Steril.

[12] Ankum WM, Mol BW, Van der Veen F, Bossuyt PM (1996). Risk factors for ectopic pregnancy: a meta-analysis. Fertil Steril.

[13] Noh J-W, Kwon YD, Yang Y, Cheon J, Kim J (2018). Relationship between body image and weight status in east Asian countries: comparison between South Korea and Taiwan. BMC Public Health.

[14] Fu T, Wang J, Xu S, Yu J, Sun G (2022). Media internalized pressure and restrained eating Behavior in College students: the multiple Mediating effects of Body Esteem and Social Physique anxiety. Front Psychol.

[15] Cai J, Liu L, Jiang X, Li P, Sha A, Ren J (2021). Low body mass index is associated with ectopic pregnancy following assisted reproductive techniques: a retrospective study. BJOG.

[16] Pan Y, Zhang S, Wang Q, Shen H, Zhang Y, Li Y, Yan D, Sun L (2016). Investigating the association between prepregnancy body mass index and adverse pregnancy outcomes: a large cohort study of 536 098 Chinese pregnant women in rural China. BMJ Open.

[17] Roth D, Grazi RV, Lobel SM (2003). Extremes of body mass index do not affect first-trimester pregnancy outcome in patients with infertility. Am J Obstet Gynecol.

[18] Marchbanks PA, Annegers JF, Coulam CB, Strathy JH, Kurland LT (1988). Risk factors for ectopic pregnancy. A population-based study. JAMA.

[19] Li C, Zhao W-H, Zhu Q, Cao S-J, Ping H, Xi X, Qin G-J, Yan M-X, Zhang D, Qiu J (2015). Risk factors for ectopic pregnancy: a multi-center case-control study. BMC Pregnancy Childbirth.

[20] Elgemark K, Graner S, McTaggart J, Ramirez Löfström J, Sörensen D, Envall N, Kopp Kallner H (2022). The 13.5-mg, 19.5-mg, and 52-mg levonorgestrel-releasing Intrauterine systems and risk of ectopic pregnancy. Obstet Gynecol.

[21] Haggerty CL, Gottlieb SL, Taylor BD, Low N, Xu F, Ness RB (2010). Risk of sequelae after Chlamydia trachomatis genital infection in women. J Infect Dis.

[22] Hoenderboom BM, van Benthem BHB, van Bergen JEAM, Dukers-Muijrers NHTM, Götz HM, Hoebe CJPA, Hogewoning AA, Land JA, van der Sande MAB, Morré SA (2019). Relation between infection and pelvic inflammatory disease, ectopic pregnancy and tubal factor infertility in a Dutch cohort of women previously tested for chlamydia in a chlamydia screening trial. Sex Transm Infect.

[23] Thirunavuk Arasoo VJ, Masalamani M, Ramadas A, Dominic NA, Liew DD, Sia RWJ, Wanigaratne A, Weerawarna K, Wong WLL, Jeganathan R. Association between Chlamydial Infection with ectopic and full-term pregnancies: a case-control study. Trop Med Infect Dis 2022, 7(10). 10.3390/tropicalmed7100285 PMC9609621 36288026

[24] Elraiyah T, Hashim Y, Elamin M, Erwin PJ, Zarroug AE. The effect of appendectomy in future tubal infertility and ectopic pregnancy: a systematic review and meta-analysis. J Surg Res 2014, 192(2). 10.1016/j.jss.2014.08.017 25303785

[25] Bowman ZS, Smith KR, Silver RM (2015). Cesarean delivery and risk for subsequent ectopic pregnancy. Am J Perinatol.

[26] Tough SC, Newburn-Cook C, Johnston DW, Svenson LW, Rose S, Belik J (2002). Delayed childbearing and its impact on population rate changes in lower birth weight, multiple birth, and preterm delivery. Pediatrics.

[27] Mills M, Rindfuss RR, McDonald P, te Velde E (2011). Why do people postpone parenthood? Reasons and social policy incentives. Hum Reprod Update.

[28] Xu H, Lin G, Xue L, Wu W, Ding J, Liu C (2022). Ectopic pregnancy in China during 2011–2020: a single-centre retrospective study of 9499 cases. BMC Pregnancy Childbirth.

[29] Correa-de-Araujo R, Yoon SSS (2021). Clinical outcomes in high-risk pregnancies due to Advanced maternal age. J Womens Health (Larchmt).

[30] Nybo Andersen AM, Wohlfahrt J, Christens P, Olsen J, Melbye M (2000). Maternal age and fetal loss: population based register linkage study. BMJ.

[31] Kang L, Liu J, Ma Q, Jing W, Wu Y, Zhang S, Liu M (2022). Prevalence of induced abortion among Chinese women aged 18–49 years: findings from three cross-sectional studies. Front Public Health.

[32] Luo H, Wu S, Wang K, Xu J, Tang L, Temmerman M, Zhang W-H (2021). Repeat induced abortion in 30 Chinese provinces: a cross-sectional study. Int J Gynaecol Obstet.

[33] Tharaux-Deneux C, Bouyer J, Job-Spira N, Coste J, Spira A (1998). Risk of ectopic pregnancy and previous induced abortion. Am J Public Health.

[34] Skjeldestad FE, Gargiullo PM, Kendrick JS (1997). Multiple induced abortions as risk factor for ectopic pregnancy. A prospective study. Acta Obstet Gynecol Scand.

[35] Martin B, Pearson M, Kebejian L, Golden E, Keselman A, Bender M, Carlson O, Egan J, Ladenheim B, Cadet J-L (2007). Sex-dependent metabolic, neuroendocrine, and cognitive responses to dietary energy restriction and excess. Endocrinology.

[36] Fowke JH, Matthews CE, Yu H, Cai Q, Cohen S, Buchowski MS, Zheng W, Blot WJ (2010). Racial differences in the association between body mass index and serum IGF1, IGF2, and IGFBP3. Endocr Relat Cancer.

[37] Popovic V, Casanueva FF (2002). Leptin, nutrition and reproduction: new insights. Horm (Athens).

[38] Hess AP, Talbi S, Hamilton AE, Baston-Buest DM, Nyegaard M, Irwin JC, Barragan F, Kruessel JS, Germeyer A, Giudice LC (2013). The human oviduct transcriptome reveals an anti-inflammatory, anti-angiogenic, secretory and matrix-stable environment during embryo transit. Reprod Biomed Online.

